# Erythropoietin modulates hepatic inflammation, glucose homeostasis, and soluble epoxide hydrolase and epoxides in high‐fat diet‐induced obese mice

**DOI:** 10.1002/2211-5463.70208

**Published:** 2026-01-31

**Authors:** Takeshi Goda, Satoru Sugimoto, Chiharu Cho, Madoka Konishi, Nozomi Inoue, Satoshi Miyagaki, Yasuhiro Kawabe, Takuro Okamura, Masahide Hamaguchi, Hisakazu Nakajima, Michiaki Fukui, Masakazu Shinohara, Tomoko Iehara

**Affiliations:** ^1^ Department of Pediatrics, Graduate School of Medical Science Kyoto Prefectural University of Medicine Japan; ^2^ Department of Endocrinology and Metabolism, Graduate School of Medical Science Kyoto Prefectural University of Medicine Japan; ^3^ The Integrated Center for Mass Spectrometry Kobe University Graduate School of Medicine Japan

**Keywords:** CYP epoxygenase‐derived epoxides, erythropoietin, hepatic inflammation, lipid mediators, obesity, soluble epoxide hydrolase

## Abstract

Obesity‐related liver disease remains a critical global health challenge, underscoring the need to elucidate the molecular mechanisms underlying hepatic inflammation and metabolic dysfunction, and to identify novel therapeutic targets. This study aimed to determine whether erythropoietin (EPO) modulates soluble epoxide hydrolase (sEH) and lipid mediator pathways to ameliorate hepatic inflammation and metabolic dysfunction in high‐fat diet (HFD)‐induced obese mice. Male C57BL/6 mice were fed HFD with or without EPO treatment, and metabolic phenotyping, including glucose tolerance testing and homeostasis model assessment of insulin resistance, was performed. Hepatic histology and quantitative real‐time polymerase chain reaction were conducted, together with flow cytometry to assess macrophage polarization, western blotting for sEH, and targeted liquid chromatography–tandem mass spectrometry profiling of cytochrome P450 epoxygenase‐derived epoxides. EPO treatment improved glucose metabolism, reduced hepatic steatosis, lowered *Ccr2*, *Mcp1*, and *Tnfα* expression, promoted a shift of hepatic macrophages toward an M2 (anti‐inflammatory) phenotype, downregulated hepatic sEH protein levels, and increased both hepatic and plasma concentrations of epoxygenase‐derived epoxides. These findings indicate that EPO suppresses hepatic sEH and favors pro‐resolving lipid mediator signaling, suggesting a potential therapeutic avenue for obesity‐related hepatic inflammation.

## Graphical abstract

Erythropoietin administration suppresses hepatic soluble epoxide hydrolase (sEH) expression, leading to increased CYP‐derived epoxides. This is associated with a shift in hepatic macrophage polarization characterized by reduced M1 markers and increased M2 markers, along with reduced hepatic inflammation, suppressed hepatic lipogenesis, and attenuated hepatic steatosis in high‐fat diet‐induced obesity. This graphic abstract was created using BioRender.com.

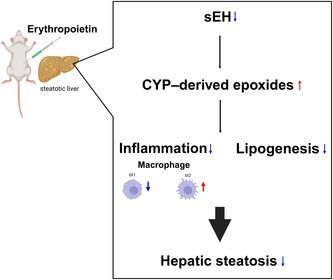

**Abbreviations**
AA: arachidonic acid
COX: cyclooxygenase
CYP: cytochrome P450 monooxygenase
DHA: docosahexaenoic acid
DHETs: dihydroxyeicosatrienoic acids
DiHDPEs: dihydroxydocosapentaenoic acids
DiHETEs: dihydroxyeicosatetraenoic acids
EETs: epoxyeicosatrienoic acids
EPA: eicosapentaenoic acid
EpDPEs: epoxydocosapentaenoic acids
EpETEs: epoxyeicosatetraenoic acids
EPO: erythropoietin
GTT: glucose tolerance test
HFD: high‐fat diet
HOMA‐IR: homeostasis model assessment of insulin resistance
LOX: lipoxygenase
MASLD: metabolic dysfunction‐associated steatotic liver disease
NAFLD: nonalcoholic fatty liver disease
PUFAs: polyunsaturated fatty acids
sEH: soluble epoxide hydrolase

Obesity is a major global health concern with long‐term metabolic consequences. One critical obesity‐related complication is metabolic dysfunction‐associated steatotic liver disease (MASLD), formerly nonalcoholic fatty liver disease (NAFLD), which contributes to insulin resistance and broader metabolic impairment [1, 2]. As obesity advances, immune cells such as macrophages, neutrophils, and lymphocytes infiltrate insulin‐responsive tissues, disrupting insulin signaling. This inflammatory environment ultimately impairs insulin sensitivity, a key characteristic of metabolic disorders. As a therapeutic approach to obesity, controlling hepatic inflammation may help ameliorate metabolic abnormalities such as insulin resistance [3, 4, 5].

EPO is a glycoprotein hormone produced in the kidneys that serves as a key regulator of erythropoiesis by driving the production of red blood cells within the bone marrow. EPO is widely administered for the management of anemia associated with chronic kidney disease and in preterm infants [6]. Recent studies indicate EPO may have additional effects beyond hematopoiesis, including anti‐obesity and anti‐inflammatory properties [7, 8, 9]. Previous studies reported that EPO may attenuate hepatic inflammation and improve insulin sensitivity in obesity model rodents, although this effect is not yet fully established [10, 11, 12, 13].

Lipid mediators are bioactive oxylipins derived from polyunsaturated fatty acids (PUFAs) such as arachidonic acid (AA), eicosapentaenoic acid (EPA), and docosahexaenoic acid (DHA). Lipid mediators exert multiple functions, including the orchestration of inflammatory initiation and resolution, and broader roles in the pathogenesis of metabolic and neurodegenerative disorders. Cyclooxygenase (COX), lipoxygenase (LOX), and cytochrome P450 monooxygenase (CYP) pathways regulate lipid mediators [13, 14, 15]. The imbalance of these lipid mediators contributes to chronic inflammatory pathologies, including obesity‐related liver disease. Targeting lipid mediators represents a promising therapeutic approach for obesity‐associated inflammation, as such strategies are less likely than steroidal or nonsteroidal anti‐inflammatory drugs (NSAIDs) to cause adverse effects, including broad immunosuppression [14, 15].

sEH, encoded by epoxide hydrolase 2 (*Ephx2*), is a key enzyme that converts CYP epoxygenase‐derived epoxides, such as epoxyeicosatrienoic acids (EETs), epoxyeicosatetraenoic acids (EpETEs), and epoxydocosapentaenoic acids (EpDPEs), into their less active diol derivatives, including dihydroxyeicosatrienoic acids (DHETs), dihydroxyeicosatetraenoic acids (DiHETEs), and dihydroxydocosapentaenoic acids (DiHDPEs). These epoxides exert potent anti‐inflammatory, vasodilatory, and insulin‐sensitizing effects [16, 17, 18]. In obesity‐related liver disease, increased *Ephx2* expression accelerates epoxide degradation, reducing their protective benefits [19, 20]. Recent studies indicate that pharmacological inhibition of sEH increases epoxide levels, leading to reduced hepatic inflammation, improved insulin sensitivity, and decreased hepatic lipid accumulation [21, 22]. Thus, modulation of *Ephx2* expression in the liver represents a promising therapeutic strategy to improve hepatic metabolism.

This study investigated the involvement of sEH in mediating the metabolic and anti‐inflammatory effects of EPO in the liver of high‐fat diet‐induced obese mice. Our results suggest that EPO improved glucose tolerance and insulin sensitivity and reduced hepatic inflammation. Moreover, EPO suppresses sEH expression and increases CYP epoxygenase‐derived epoxides levels, which confer anti‐inflammatory and metabolic benefits. These findings highlight the therapeutic potential of EPO for managing obesity‐related metabolic dysfunction and hepatic inflammation.

## Materials and methods

### Animal experimental procedure and ethics statement

Four‐week‐old specific‐pathogen‐free male C57BL/6 mice were purchased from CLEA Japan (Tokyo, Japan) and housed in a temperature‐controlled room at 24 °C on a 12‐h light/dark cycle, with free access to food and water unless indicated. Mice were fed a HFD (60 kcal% fat; Research Diets, New Brunswick, NJ, USA) for 7 weeks. Body weight and food intake were monitored weekly and every other day, respectively. Food intake was measured per cage, and the average food consumption per mouse was calculated for each group. HFD‐fed mice were randomly assigned to groups and received intraperitoneally (i.p.) with 300 IU·kg^−1^ of recombinant human EPO (Epoetin Alfa BS injection; JCR Pharmaceuticals Co., Ltd., Ashiya, Japan) (HFE group, *n* = 12) or with saline (HF group, *n* = 12) three times a week for 7 weeks. At week 11, mice were randomly selected from each group and euthanized for collection of liver and blood. Mice were anesthetized with isoflurane (Pfizer, Tokyo, Japan) inhalation, after which blood was obtained via cardiac puncture. Plasma was separated via centrifugation at 4 °C and stored at −80 °C until further analysis. Livers were excised, weighed, rapidly frozen in liquid nitrogen, and stored at −80 °C until subsequent analyses. All animal procedures were approved by the Institutional Animal Care and Use Committee of Kyoto Prefectural University of Medicine (approval no. M2024‐40) and complied with national regulations.

### Glucose tolerance test

For the glucose tolerance test (GTT), the animals were fasted overnight. Fasting glucose levels were assessed using a drop of blood from the tail tip. Glucose was administered intraperitoneally at a dose of 2 g·kg^−1^ of body weight. Blood glucose concentrations were subsequently determined at 15, 30, 60, 90, and 120 min following injection. Blood glucose levels were measured using a Glutest neo sensor® (Sanwa Kagaku Kenkyusho, Nagoya, Japan).

### Homeostasis model assessment of insulin resistance

An ELISA kit was used to measure plasma insulin levels (Morinaga Institute of Biological Science, Kanagawa, Japan). The homeostasis model assessment of insulin resistance (HOMA‐IR) was calculated based on fasting plasma glucose level (mg·dL^−1^) and insulin level (μU·mL^−1^) using the following equation: HOMA‐IR = (glucose × insulin)/405.

### Histology and oil red O staining

Liver tissue was processed by the Institute of Nutrition and Pathology (Tokyo, Japan). Frozen liver sections were prepared using a cryostat and mounted on glass slides. An Oil Red O stock solution (0.25 g/100 mL in isopropanol) was prepared, then heated to 100 °C for 10 min. Liver sections were fixed in 4% paraformaldehyde for 30 min and subsequently washed with PBS. A 60% working solution of Oil Red O was prepared by diluting the stock solution with distilled water, and the sections were incubated in this solution for 30 min. Following staining, the sections were rinsed with PBS until the background was clear. Images were acquired using a BZ‐X810 fluorescence microscope (Keyence, Osaka, Japan).

### Quantitative real‐time polymerase chain reaction (qRT‐PCR)

To analyze mRNA expression, total RNA was isolated from the liver using a NucleoSpin RNA II kit (cat. no. 740955.50; Macherey‐Nagel, Düren, Germany). Template cDNA was synthesized from 1 μg of total RNA using random hexamer primers for each reaction in a ReverTra Ace qPCR RT Master Mix (cat. no. FSQ201; Toyobo, Osaka, Japan). We performed qRT‐PCR as previously described [23]. The mRNA expression levels of target genes were quantified by qRT‐PCR using TB Green Premix Ex Taq II (Tli RNaseH Plus; Takara, Shiga, Japan). Fluorescence signals were detected with an AB 7500 Real‐Time PCR System (Applied Biosystems, Tokyo, Japan). Relative mRNA expression was calculated by the comparative ΔΔ*C*_t_ method using β‐actin as the housekeeping gene. The primers used in this study are shown in Table S1.

### Western blot analysis

Liver proteins were extracted using a radioimmunoprecipitation assay (RIPA) lysis buffer (cat. no. 08714.04; Nacalai Tesque, Kyoto, Japan). The homogenates were centrifuged at 10 000 ***g*** for 10 min at 4 °C, and the resulting supernatants were collected. Protein concentrations were determined using a Protein Quantification Assay Kit (cat. no. 740967.50; Macherey‐Nagel). Extracted proteins were separated into 10% polyacrylamide gels with sodium dodecyl sulfate and transferred to polyvinylidene difluoride membranes. Membranes were blocked using Bullet Blocking One for Western Blotting (cat. no. 13779‐14; Nacalai Tesque). Primary and secondary antibodies were diluted with Can‐Get Signal (cat. no. NKB‐101; Toyobo, Osaka, Japan). The membrane was washed and incubated with primary antibodies against sEH (1 : 100) (Santa Cruz Biotechnology, Dallas, TX, USA) and β‐actin (1 : 1000) (Cell Signaling Technology, Danvers, MA, USA). The secondary antibody, HRP‐conjugated sheep anti‐mouse IgG (GE Healthcare), was applied at a 1 : 15 000 dilution. Immunocomplexes were visualized using an enhanced HRP–luminol chemiluminescence detection system (ECL Prime; GE Healthcare, Chicago, IL, USA) and subsequently exposed to autoradiography film (New Amersham Hyperfilm; GE Healthcare). The intensity of the immunoblot signals was quantified from raw images using imagej software (version 1.54; National Institutes of Health, Bethesda, MD, USA).

### Protocols for isolation of mononuclear cells from liver

Isolation of mononuclear cells from liver was performed as described previously [24]. The excised liver tissue was gently dissociated by passing it through a 70‐μm cell strainer, and the resulting cell suspension was prepared in Roswell Park Memorial Institute (RPMI) 1640 medium supplemented with 2% fetal bovine serum (FBS). The suspension was centrifuged at 400 ***g***, and the resulting pellet was resuspended in 40% Percoll solution. This suspension was layered over an equal volume of 60% Percoll solution and centrifuged at 700 ***g*** for 20 min at room temperature. Cells collected from the Percoll interface (buffy coat) were subsequently pelleted by centrifugation and washed twice with PBS containing 2% FBS prior to use.

### Flow cytometry

Flow cytometry was performed as described previously [24]. Data acquisition and analysis were performed using a FACS Canto II flow cytometer and flowjo software (version 10; TreeStar, Ashland, OR, USA). M1 and M2 macrophage populations were identified by flow cytometric gating based on the following antibodies: APC‐conjugated CD45.2 (17045482; clone: 104; 1 : 50; eBioscience, San Diego, CA, USA), APC‐Cy7‐conjugated CD11b (47011282; clone: M1/70; 1 : 50; eBioscience), PE‐conjugated F4/80 (12480182; clone: BM8; 1 : 50; eBioscience), FITC‐conjugated CD206 (MA516870; clone: MR5D3; 1 : 50; eBioscience), and PE‐Cy7‐conjugated CD11c (25011482; clone: N418; 1 : 50; eBioscience) [25] (Fig. S1). M1 macrophages were defined as CD45^+^ F4/80^+^ CD206^−^ CD11c^+^ cells, whereas M2 macrophages were defined as CD45^+^ F4/80^+^ CD206^+^ CD11c^−^ cells.

### LC–MS/MS analysis

Deuterated internal standards (d_4_‐leukotriene B_4_, d_8_‐5‐hydroxyeicosatetraenoic acid, d_4_‐prostaglandin E_2_, and d_5_‐resolvin D_2_) each representing a distinct chromatographic region of the targeted lipid mediators, were added to the samples at a concentration of 500 pg per sample to enable accurate quantification. Lipid mediators were extracted by solid‐phase extraction using C18 columns as previously described [26] and subsequently analyzed by LC–MS/MS with a Qtrap 6500 mass spectrometer (Sciex, Tokyo, Japan) coupled to an LC‐30AD high‐performance liquid chromatography system (Shimadzu, Kyoto, Japan). Separation was achieved on a ZORBAX Eclipse Plus C18 column (100 × 4.6 mm, 3.5 μm; Agilent Technologies, Santa Clara, CA, USA) using a methanol/water/acetic acid gradient from 55 : 45 : 0.01 (v/v/v) to 98 : 2 : 0.01 at a flow rate of 0.4 mL·min^−1^. For targeted lipid mediator detection and quantification, a multiple reaction monitoring (MRM) method was established with specific Q1 (parent ion) and Q3 (fragment ion) transitions defined for each compound. Identification of individual lipid mediators was confirmed based on LC retention time, characteristic fragmentation patterns, and the presence of at least six diagnostic fragment ions, as described previously. Quantification was performed by calculating the peak area in the MRM chromatograms, with linear calibration curves generated using authentic standards for each analyte.

### Statistical analysis

Data are expressed as the mean ± SEM. Statistical analysis was performed using graphpad prism version 10.6 software (GraphPad Software, LLC, San Diego, CA, USA). Differences between two groups were analyzed using unpaired two‐tailed Student's *t*‐tests. Repeated measures data, including body weight gain and GTT, were evaluated by two‐way repeated measures ANOVA followed by Bonferroni correction. *P* < 0.05 was considered statistically significant.

## Results

### EPO administration suppressed weight gain and improved glucose metabolism in high‐fat diet‐induced obese mice

**Fig. 1:**
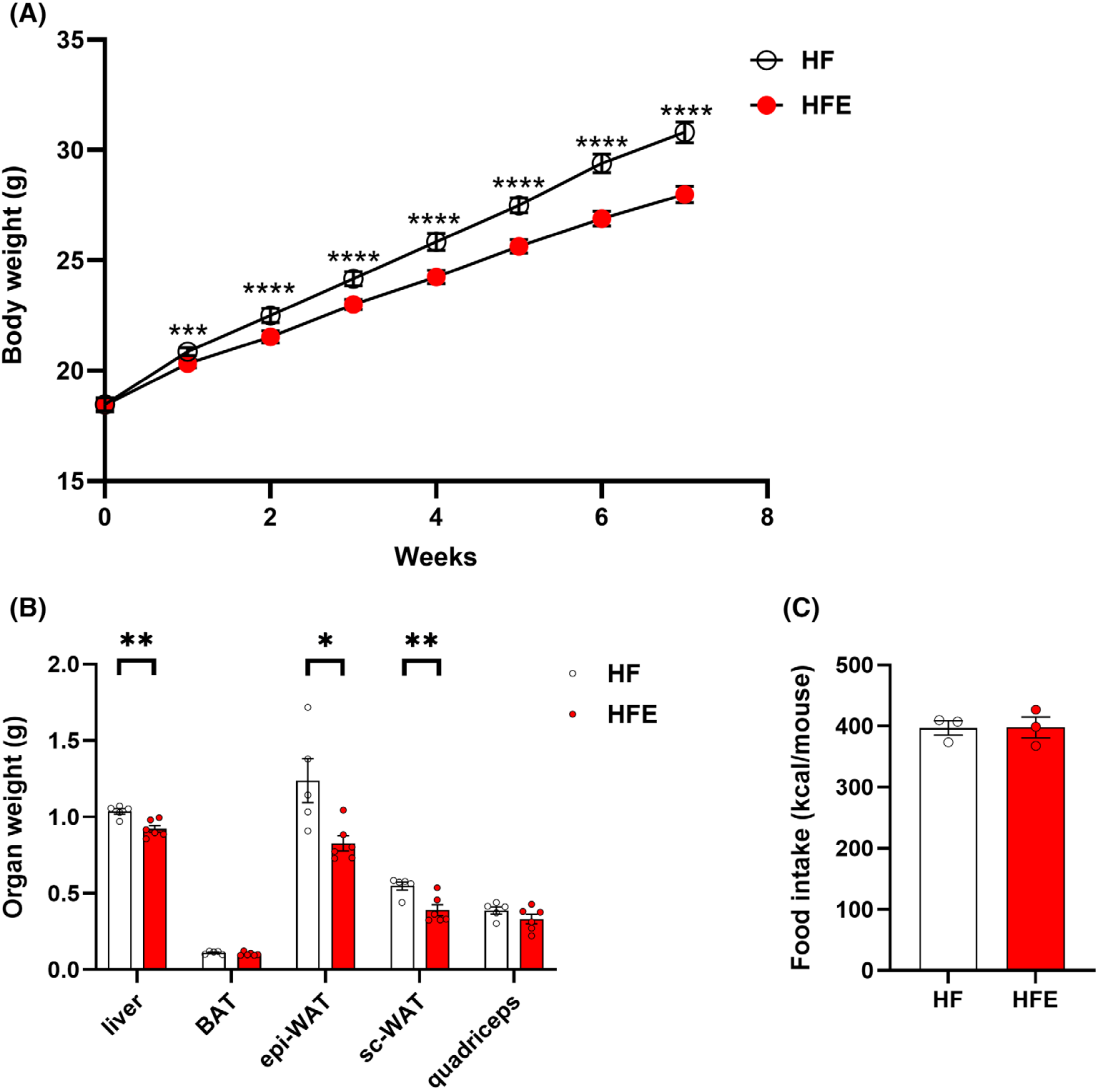
Effects of erythropoietin (EPO) administration on body weight, organ weight, and food intake. Male C57BL/6J mice (4 weeks old) were fed a high‐fat diet for 7 weeks. EPO group (HFE) received EPO (300 IU·kg^−1^, intraperitoneally, three times/week). (A) Weekly change in body weight. Values are mean ± SEM; HF (*n* = 12) and HFE (*n* = 12) mice. Differences between groups over time were analyzed using two‐way repeated measures ANOVA followed by Bonferroni's multiple comparisons test. *P < 0.05, **P < 0.01, ***P < 0.001, ****P < 0.0001. (B) Organ weight. Values are mean ± SEM; HF (*n* = 5) and HFE (*n* = 6) mice. Differences between two groups were analyzed using unpaired two‐tailed Student's *t*‐tests. *P < 0.05, **P < 0.01. (C) Total food intake. Values are mean ± SEM; HF (*n* = 3) and HFE (*n* = 3) in separate cages. Differences between two groups were analyzed using unpaired two‐tailed Student's *t*‐tests. BAT, brown adipose tissue; epi‐WAT, epididymal white adipose tissue; sc‐WAT, subcutaneous white adipose tissue.

**Fig. 2:**
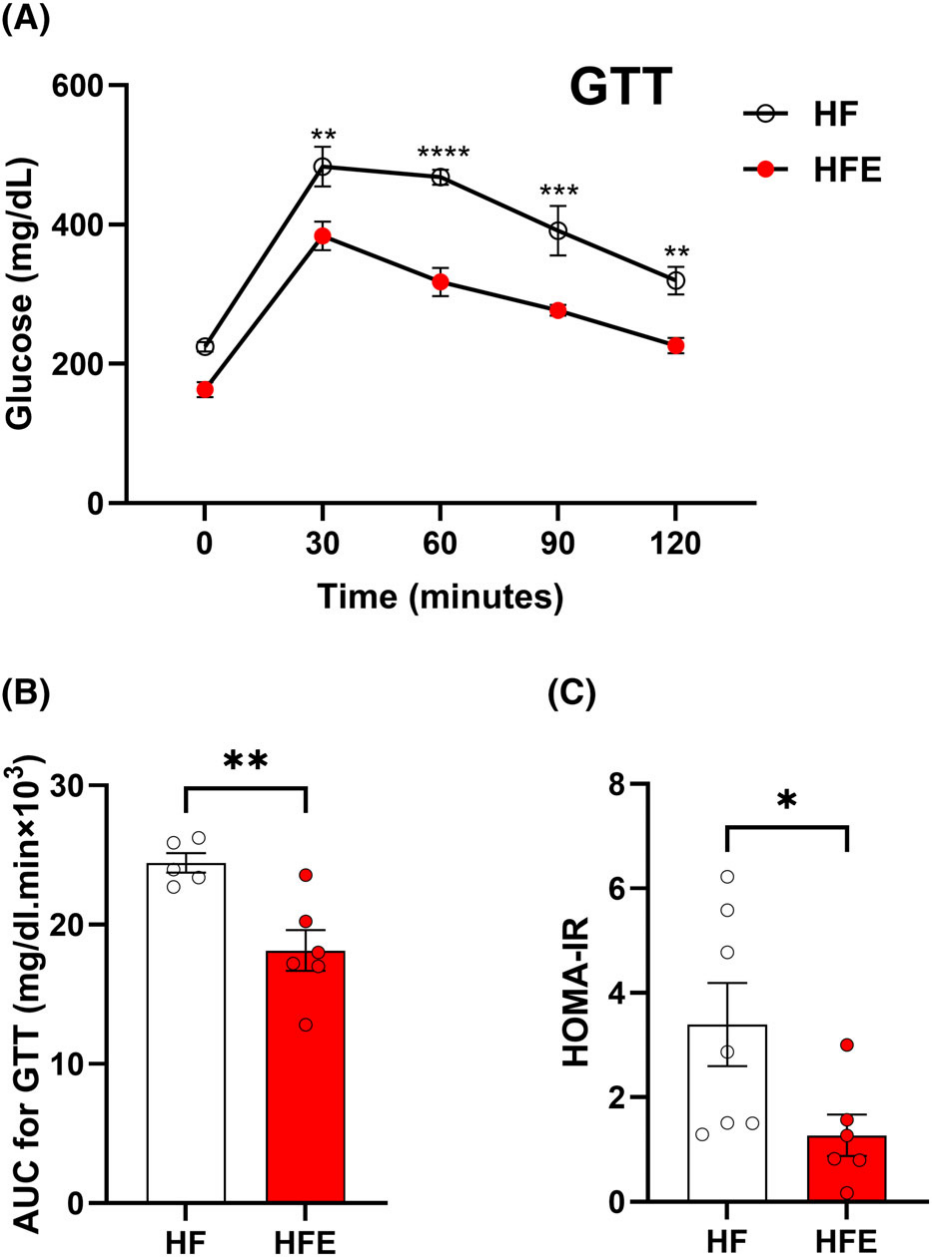
Erythropoietin (EPO) improves glucose tolerance and insulin sensitivity. GTT was performed after an overnight fast, followed by intraperitoneal injection of glucose (2 g·kg^−1^). (A) Glucose tolerance test (GTT). Values are mean ± SEM; HF (*n* = 5) and HFE (*n* = 6) mice. Differences between groups over time were analyzed using two‐way repeated measures ANOVA followed by Bonferroni's multiple comparisons test. *P < 0.05, **P < 0.01, ***P < 0.001, ****P < 0.0001. (B) Area under the curve (AUC) for GTT. Values are mean ± SEM; HF (*n* = 5) and HFE (*n* = 6) mice. Differences between two groups were analyzed using unpaired two‐tailed Student's *t*‐tests. *P < 0.05, **P < 0.01. (C) HOMA‐IR. Values are mean ± SEM; HF (*n* = 5) and HFE (*n* = 6) mice. Differences between two groups were analyzed using unpaired two‐tailed Student's *t*‐tests. *P < 0.05.

After 7 weeks, the HFE group mice had significantly lower body weight than the HF group mice (Fig. 1A). Organ weight analysis showed significant reductions in liver weight, epididymal white adipose tissue mass, and subcutaneous white adipose tissue mass in the HFE group compared with the HF group (Fig. 1B). No significant differences in food intake were observed between the two groups (Fig. 1C). Glucose intolerance and insulin sensitivity were significantly improved by EPO administration (Fig. 2A–C).

### EPO suppressed lipid accumulation in the liver of high‐fat diet‐induced obese mice

**Fig. 3:**
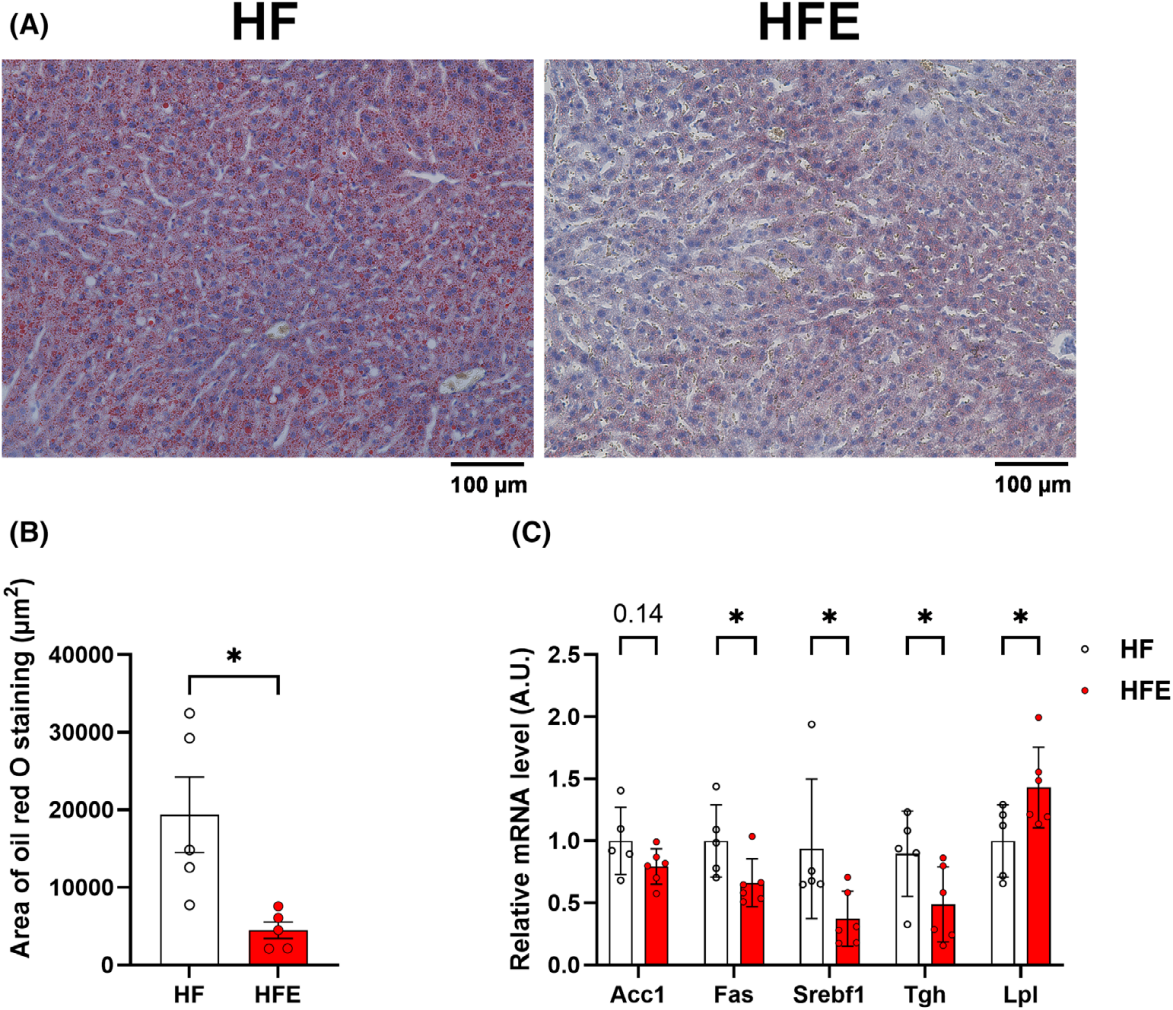
Suppression of hepatic lipid accumulation by erythropoietin (EPO). (A) Representative photomicrographs of liver sections stained with Oil Red O. Scale bars = 100 μm. (B) The area of Oil Red O staining was quantified using imagej. Values are mean ± SEM; HF (*n* = 5) and HFE (*n* = 5) mice. Differences between two groups were analyzed using unpaired two‐tailed Student's *t*‐tests. *P < 0.05. (C) Real‐time PCR experiments for lipogenesis and lipolysis markers (*Acc1*, *Fas*, *Tgh*, *Srebf1*, *Lpl*) were normalized to β‐actin. A.U., arbitrary units. Values are mean ± SEM; HF (*n* = 5) and HFE (*n* = 6) mice. Differences between two groups were analyzed using unpaired two‐tailed Student's *t*‐tests. *P < 0.05.

Oil Red O staining of liver tissue revealed prominent lipid accumulation in the HF group mice, which was attenuated in HFE group mice (Fig. 3A,B). EPO significantly decreased expression of lipogenic gene, acetyl‐CoA carboxylase 1 (*Acc1*), fatty acid synthase (*Fas*), triacylglycerol hydrolase (*Tgh*), and sterol regulatory element‐binding transcription factor 1 (*Srebf1*), and increased expression of lipolytic genes, lipoprotein lipase (*Lpl*) in HFE group mice compared with HF group mice (Fig. 3C). These findings suggest that EPO attenuated hepatic lipid accumulation at least in part by suppressing *de novo* lipogenesis.

### EPO regulated the macrophage phenotype and ameliorated inflammation in the liver of high‐fat diet‐induced obese mice

**Fig. 4:**
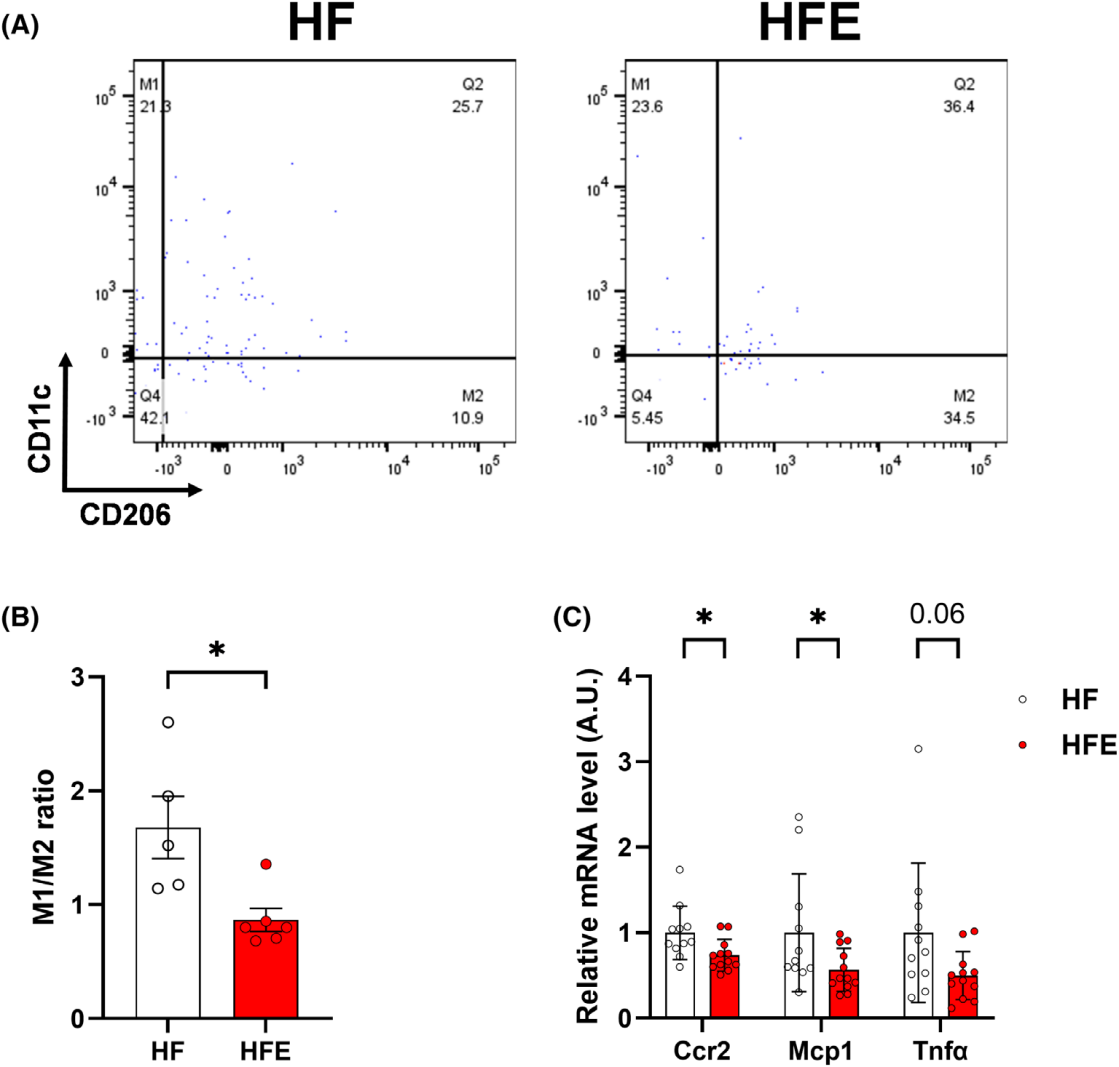
Modulation of macrophage polarization and inflammatory gene expression by erythropoietin (EPO). (A) Representative flow cytometry profiles showing liver CD45.2^+^ F4/80^+^ CD206^−^ CD11c^+^ M1 macrophages and CD45.2^+^ F4/80^+^ CD206^+^ CD11c^−^ M2 macrophages in each group. (B) M1/M2 ratio. Values are mean ± SEM; HF (*n* = 5) and HFE (*n* = 6) mice. Differences between two groups were analyzed using unpaired two‐tailed Student's *t*‐tests. *P < 0.05. (C) Real‐time PCR experiments for inflammation markers (*Ccr2*, *Mcp1*, *Tnfα*) were normalized to β‐actin. A.U., arbitrary units. Values are mean ± SEM; HF (*n* = 11) and HFE (*n* = 12) mice. Differences between two groups were analyzed using unpaired two‐tailed Student's *t*‐tests. *P < 0.05.

We examined changes in hepatic macrophage phenotype by flow cytometry. M1 macrophages are associated with a pro‐inflammatory response, whereas M2 macrophages are linked to anti‐inflammatory functions [27]. The hepatic M1/M2 macrophage ratio is associated with hepatic inflammation, and polarization toward the M2 phenotype contributes to the suppression of inflammation [28, 29]. The hepatic M1/M2 macrophage ratio was significantly lower in HFE group mice than in HF group mice (Fig. 4A,B). This finding indicates that EPO promoted an increase in M2 macrophages while decreasing M1 macrophages in high‐fat diet‐induced obese mice, suggesting that its anti‐inflammatory effects may be driven by macrophage polarization. Compared with HF group mice, HFE group mice exhibited significantly lower hepatic expression of the macrophage‐associated pro‐inflammatory genes, C‐C chemokine receptor type 2 (*Ccr2*), and monocyte chemoattractant protein‐1 (*Mcp1*), with a downward trend also observed for Tumor necrosis factor alpha (*Tnfα*) (Fig. 4C). These findings suggest that EPO exerts anti‐inflammatory effects by acting on macrophages.

### EPO suppressed sEH expression in the liver of high‐fat diet‐induced obese mice

**Fig. 5:**
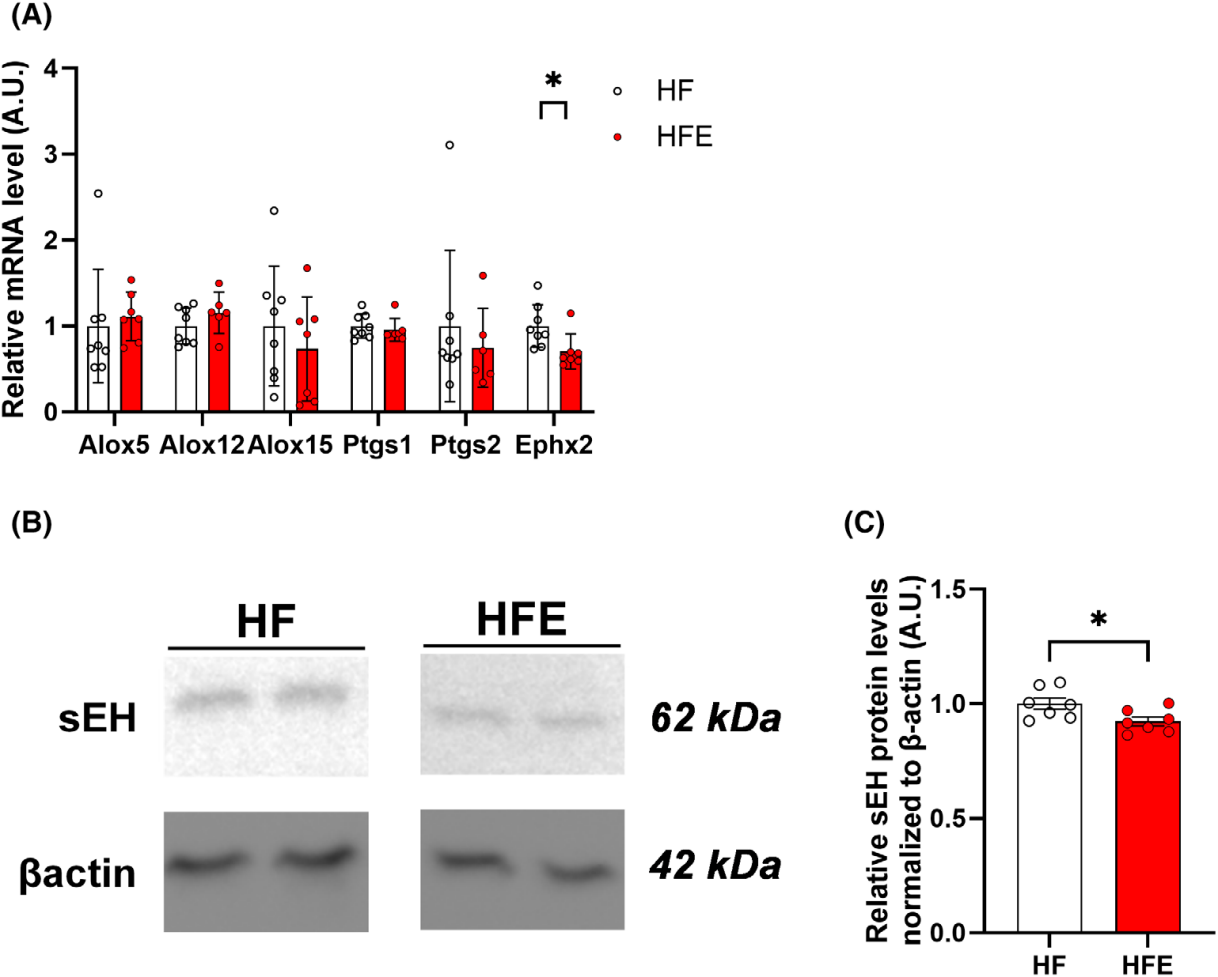
Lipid mediator‐related enzyme expression with erythropoietin (EPO). (A) Real‐time PCR experiments for lipid mediator‐related markers (*Alox5*, *Alox12*, *Alox15*, *Ptgs1*, *Ptgs2*, *Ephx2*) were normalized to β‐actin. A.U., arbitrary units. Values are mean ± SEM; HF (*n* = 8) and HFE (*n* = 6–7) mice. Differences between two groups were analyzed using unpaired two‐tailed Student's *t*‐tests. *P < 0.05. (B) Western blot analysis for sEH expression in liver tissue. Representative blots of sEH (62 kDa) and β‐actin (42 kDa) are shown. Spliced lanes from the same blot are shown. (C) sEH expression was quantified by imagej. A.U., arbitrary units. Values are mean ± SEM; HF (*n* = 7) and HFE (*n* = 7) mice. Differences between two groups were analyzed using unpaired two‐tailed Student's *t*‐tests. *P < 0.05.

Previous studies have shown that hepatic sEH protein levels are increased in high‐fat diet‐induced obese mice [21, 22, 30, 31]. In our study, EPO treatment selectively decreased the expression of *Ephx2*, whereas no significant differences were observed in arachidonate 5‐lipoxygenase (*Alox5*), arachidonate 12‐lipoxygenase (*Alox12*), arachidonate 15‐lipoxygenase (*Alox15*), prostaglandin‐endoperoxide synthase 1 (*Ptgs1*), or prostaglandin‐endoperoxide synthase 2 (*Ptgs2*) expressions between the HF and HFE groups (Fig. 5A).

We next assessed hepatic sEH protein levels by western blotting and found them to be significantly lower in HFE mice than in HF mice (Fig. 5B,C). These findings suggest that EPO reduced hepatic expression of *Ephx2*, leading to a decrease in sEH production.

### EPO increased hepatic and plasma levels of CYP epoxygenase‐derived epoxides in high‐fat diet‐induced obese mice

**Fig. 6:**
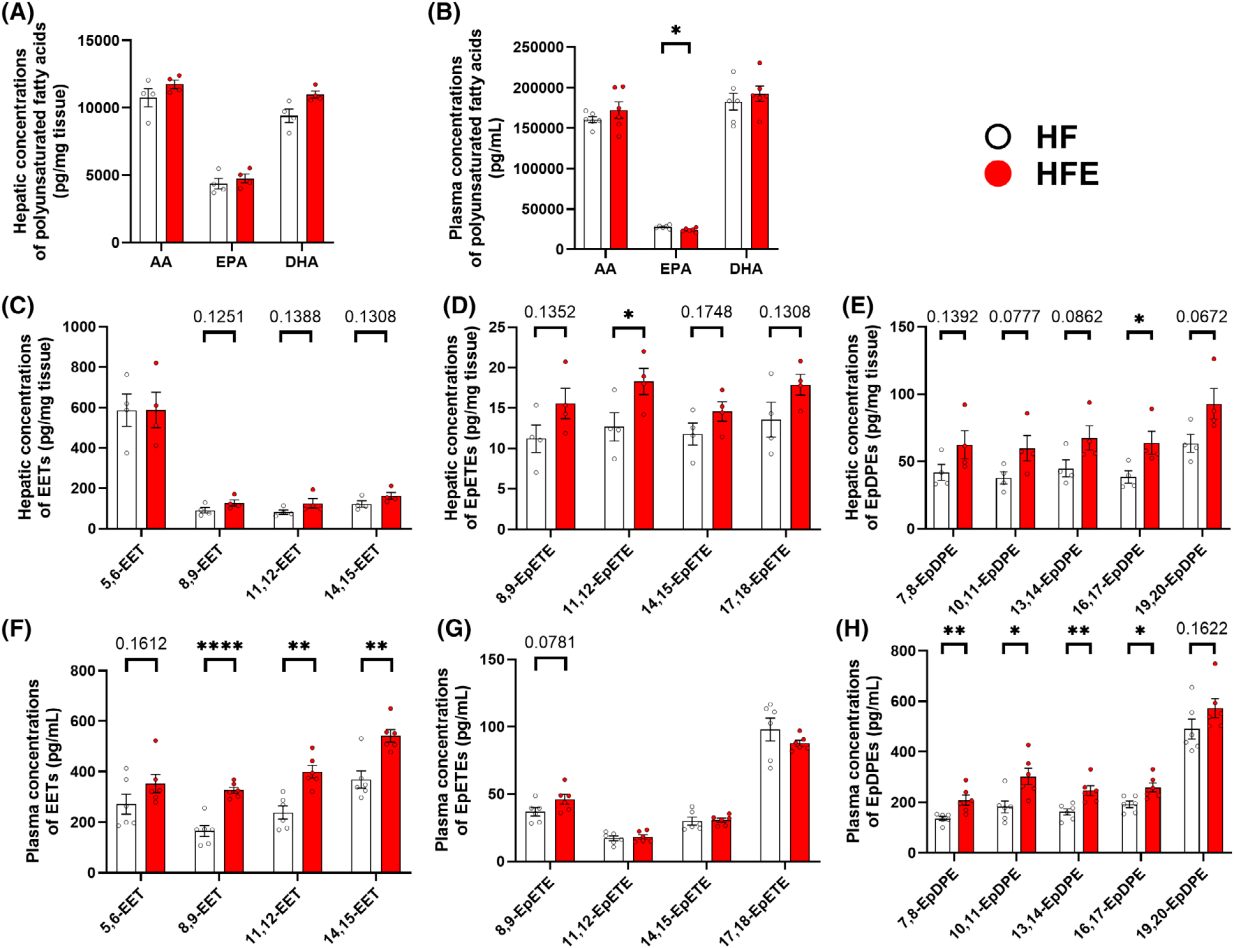
Alterations in lipid mediator profiles induced by erythropoietin (EPO). (A) Hepatic concentrations of arachidonic acid (AA), eicosapentaenoic acid (EPA), and docosahexaenoic acid (DHA). Values are mean ± SEM; HF (*n* = 4) and HFE (*n* = 4) mice. Differences between two groups were analyzed using unpaired two‐tailed Student's *t*‐tests. (B) Plasma concentrations of AA, EPA, and DHA. Values are mean ± SEM; HF (*n* = 6) and HFE (*n* = 6) mice. Differences between two groups were analyzed using unpaired two‐tailed Student's *t*‐tests. *P < 0.05. (C) Hepatic concentrations of AA‐derived epoxyeicosatrienoic acids (EETs). Values are mean ± SEM; HF (*n* = 4) and HFE (*n* = 4) mice. Differences between two groups were analyzed using unpaired two‐tailed Student's *t*‐tests. (D) Hepatic concentrations of EPA‐derived epoxyeicosatetraenoic acids (EpETEs). Values are mean ± SEM; HF (*n* = 4) and HFE (*n* = 4) mice. Differences between two groups were analyzed using unpaired two‐tailed Student's *t*‐tests. *P < 0.05. (E) Hepatic concentrations of DHA‐derived epoxydocosapentaenoic acids (EpDPEs). Values are mean ± SEM; HF (*n* = 4) and HFE (*n* = 4) mice. Differences between two groups were analyzed using unpaired two‐tailed Student's *t*‐tests. *P < 0.05. (F) Plasma concentrations of AA‐derived EETs. Values are mean ± SEM; HF (*n* = 6) and HFE (*n* = 6) mice. Differences between two groups were analyzed using unpaired two‐tailed Student's *t*‐tests. *P < 0.05, **P < 0.01, ***P < 0.001, ****P < 0.0001. (G) Plasma concentrations of EpETEs. Values are mean ± SEM; HF (*n* = 6) and HFE (*n* = 6) mice. Differences between two groups were analyzed using unpaired two‐tailed Student's *t*‐tests. (H) Plasma concentrations of EpDPEs. Values are mean ± SEM; HF (*n* = 6) and HFE (*n* = 6) mice. Differences between two groups were analyzed using unpaired two‐tailed Student's *t*‐tests. *P < 0.05, **P < 0.01.

We performed a comprehensive analysis using LC–MS/MS to assess changes in sEH‐related lipid mediators following EPO administration. There was a significant difference only in plasma levels of EPA, whereas no significant differences were observed in hepatic or plasma levels of DHA and AA between HF and HFE mice (Fig. 6A,B). With respect to hepatic concentrations of CYP epoxygenase‐derived epoxides, all species except 5,6‐EET showed a trend toward elevation in the HFE group, with 11,12‐EpETE and 16,17‐EpDPE showing significant increases (Fig. 6C–E). Plasma CYP epoxygenase‐derived epoxides, which include 8,9‐EET, 11,12‐EET, 14,15‐EET, 7,8‐EpDPE, 10,11‐EpDPE, 13,14‐EpDPE, and 16,17‐EpDPE, displayed a similar trend toward elevation in HFE mice (Fig. 6F–H). These metabolites have been reported to exert anti‐inflammatory actions [18, 32, 33], yet they are subsequently hydrolyzed by sEH into biologically less active, or even pro‐inflammatory, diol derivatives and related fatty acid diols [16, 18, 34].

## Discussion

In this study, we demonstrated that EPO suppresses sEH expression in the liver of high‐fat diet‐induced obese mice, leading to increased levels of anti‐inflammatory lipid mediators, namely CYP epoxygenase‐derived epoxides, in both circulation and liver. These findings suggest that EPO exerts anti‐inflammatory and anti‐lipogenic effects at least in part via sEH regulation. To our knowledge, there have been no previous reports establishing a connection between EPO and the function of sEH/CYP epoxygenase‐derived epoxides.

Previous studies have reported that EPO exerts protective effects against hepatic inflammation and improves glucose homeostasis in obesity [12, 35]. The underlying mechanism involves EPO‐mediated M2 macrophage polarization [36]. Consistent with these findings, our study showed that EPO administration decreased the M1/M2 macrophage ratio in the liver and increased the population of M2 macrophages, indicating that its anti‐inflammatory effects are mediated through macrophage polarization.

sEH expression and activity are upregulated in adipose tissue, liver, and kidney under high‐fat diet conditions and have been implicated in the development and progression of hepatic inflammation and steatotic liver disease [19, 31]. sEH hydrolyzes anti‐inflammatory CYP epoxygenase‐derived epoxides into less active diol metabolites [37]. CYP epoxygenase‐derived epoxides exert anti‐inflammatory, vasodilatory, and insulin‐sensitizing effects and have been shown to modulate macrophage polarization and lipid metabolism [38, 39, 40, 41]. Epoxide signaling promotes M2 polarization and suppresses pro‐inflammatory pathways such as the *Ccr2*/chemokine axis and *Tnfα* expression, thereby attenuating steatohepatitis progression [38, 39]. Consistently, pharmacological inhibition of sEH, which stabilizes these epoxides, alleviates hepatic inflammation, improves insulin resistance, and reduces lipid accumulation in obesity and alcohol‐induced liver injury models [21, 38, 39, 41, 42]. In the present study, EPO administration suppressed hepatic sEH expression, increased hepatic and circulating levels of CYP epoxygenase‐derived epoxides, promoted M2 polarization of hepatic macrophages, and reduced inflammatory and lipogenic markers. These effects were accompanied by improvements in glucose tolerance and insulin sensitivity, as evidenced by both GTT and HOMA‐IR. These findings suggest that EPO exerts anti‐inflammatory and anti‐lipogenic effects, at least in part through suppression of sEH and subsequent stabilization of CYP epoxygenase‐derived epoxides, thereby enhancing the protective actions of these lipid mediators.

A notable strength of this study is the identification of EPO, an agent already in clinical use, as a regulator of sEH expression. In contrast to previous work employing supraphysiological doses [43], our experiments used clinically relevant doses, thereby enhancing translational applicability. Although EPO can increase hematocrit, the dosage used here is comparable to neonatal clinical practice. Moreover, previous studies using sEH inhibitors have suggested that selectively inhibiting the arachidonic acid biosynthetic pathway may cause metabolic shifts to alternative pathways, potentially exacerbating serious side effects [44, 45, 46]. In this context, the finding that EPO downregulates sEH expression, rather than directly inhibiting COX/LOX, is significant, as it may help avoid serious adverse effects. Three issues merit consideration. First, while some clinical studies have reported metabolic benefits of EPO treatment in humans, no clinical investigations have specifically examined its effects on hepatic metabolism and inflammation. Second, we cannot exclude the possibility that the observed effects were partially influenced by the hematopoietic activity of EPO, including EPO‐induced increases in hematocrit. Such changes may affect circulating lipid mediator concentrations; however, the levels of AA, EPA, and DHA were not significantly altered, suggesting that any hematocrit‐related impact is likely limited. Third, locomotor activity was not evaluated. However, previous work using EPO doses and treatment durations within the therapeutic range reported no significant changes in locomotor activity after EPO treatment, making major effects on spontaneous movement unlikely [23].

In summary, we found that EPO suppresses hepatic sEH expression in high‐fat diet‐induced obese mice. These findings suggest that EPO may attenuate hepatic lipid accumulation and inflammation, at least in part through regulation of the sEH/epoxide axis, thereby offering potential therapeutic benefits for obesity‐related metabolic dysfunction.

## Conflict of interest

The authors declare no conflict of interest.

## Author contributions

TG contributed to writing—original draft, visualization, investigation, formal analysis, data curation, methodology, and conceptualization. SS contributed to writing—review and editing, methodology, conceptualization, validation, supervision, project administration, and funding acquisition. CC and MK contributed to investigation. NI contributed to investigation and methodology. SM contributed to investigation and funding acquisition. YK and HN contributed to supervision. TO and MH contributed to methodology and resources. MF contributed to supervision and resources. MS contributed to methodology, investigation, resources, and supervision. TI contributed to supervision, resources, and funding acquisition. All authors have read and approved the final version of the manuscript.

## Supporting information


**Fig. S1.** Gating strategy for flow cytometry analysis of hepatic macrophages.

**Table S1.** List of primer sequences used for real time PCR.

## Data Availability

Data supporting the findings of this study are available from the corresponding author upon reasonable request. The processed datasets and analysis scripts generated during the current study are available in Figshare at https://doi.org/10.6084/m9.figshare.31125463.
