# Supplementary figures and images for: Erythropoietin modulates hepatic inflammation, glucose homeostasis, and soluble epoxide hydrolase and epoxides in high‐fat diet‐induced obese mice
Source: FEBS Open Bio. 2026 Jan 31;16(7):1328–39. doi: 10.1002/2211-5463.70208 (PMC13327047; doi:10.1002/2211-5463.70208)

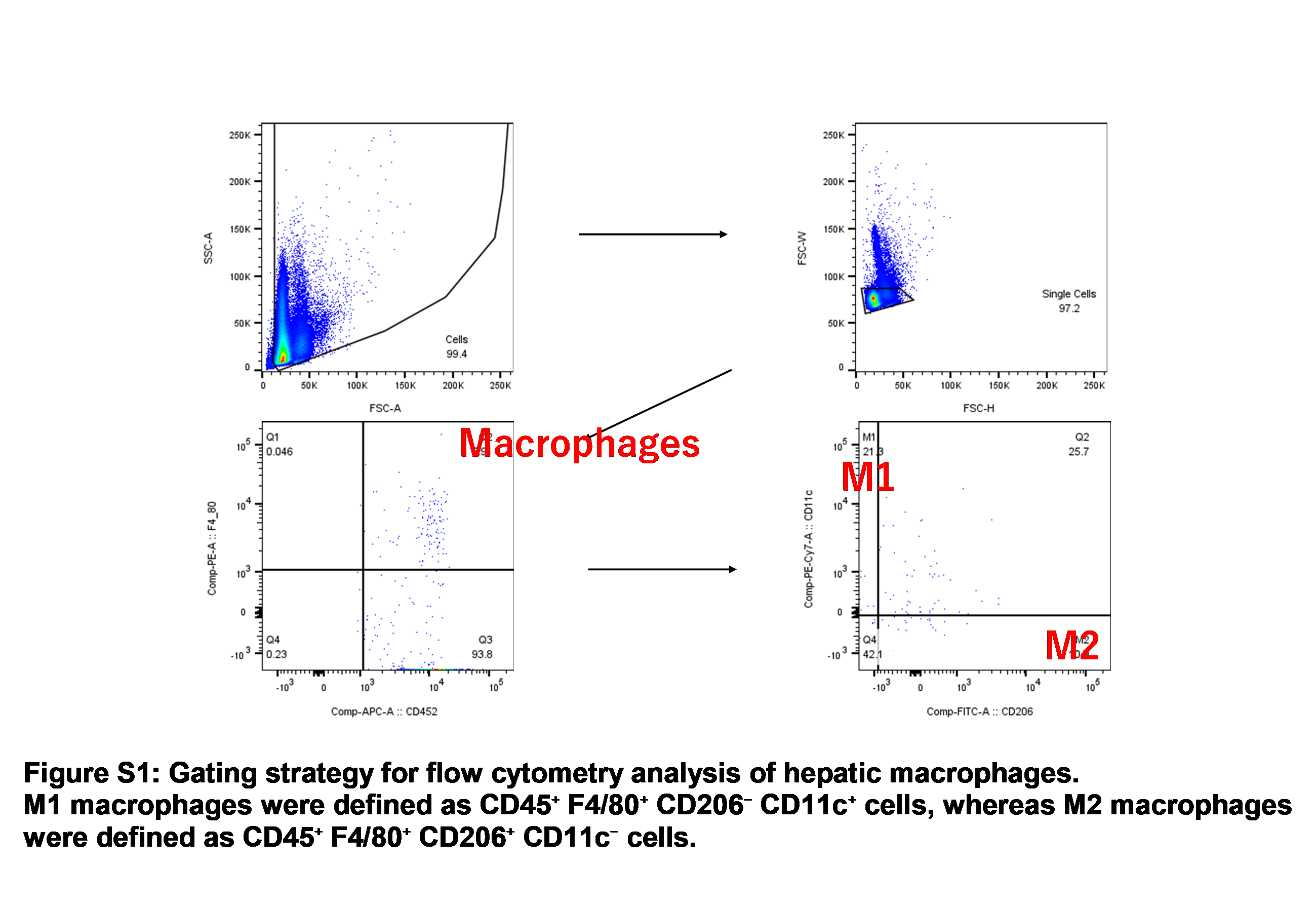

Supplement: Supplementary file 1 — Fig. S1. Gating strategy for flow cytometry analysis of hepatic macrophages. [file FEB4-16-1328-s001.tif]
